# Acute colonic pseudo-obstruction and rapid septic progression after transabdominal preperitoneal hernia repair: a case report

**DOI:** 10.1186/s12893-021-01199-y

**Published:** 2021-04-12

**Authors:** Yuki Inagaki, Kohei Matsuo, Yoritaka Nakano, Tadashi Kondo

**Affiliations:** 1 Department of Gastroenterological Surgery, Tsukuba Medical Center Hospital, 1-3-1, Amakubo, Tsukuba, Ibaraki Japan; 2 Division of Surgery, Kenpoku Medical Center, Takahagi Kyodo Hospital, 1006-9, Agehocho, Kamitezuna, Takahagi, Ibaraki Japan; 3 Division of Gastroenterological Surgery, Hitachinaka General Hospital, 20-1, Ishikawacho, Hitachinaka, Ibaraki Japan

**Keywords:** Acute colonic pseudo-obstruction, Ogilvie’s syndrome, Obstructive colitis, Laparoscopic transabdominal preperitoneal hernia repair, Case report

## Abstract

**Background:**

Acute colonic pseudo-obstruction (ACPO) is a rare condition observed in patients with some underlying medical or surgical conditions. To the best of our knowledge, this is the first case report of a patient with ACPO development and rapid septic progression after laparoscopic inguinal hernia repair.

**Case presentation:**

A 78-year-old man who underwent transabdominal preperitoneal hernia repair (TAPP) for right inguinal hernia presented with difficulty in defecation and abdominal distension. He visited our emergency department on the third postoperative day. Enhanced computed tomography (CT) detected marked enlargement from the cecum to the rectum. There was no evidence of mechanical obstruction, ischemia, or perforation. He was diagnosed with postoperative constipation and received conservative management. He gradually started to improve; however, he suddenly experienced cardiopulmonary arrest 30 h after admission and could not be resuscitated. CT imaging of the abdomen during autopsy did not show any significant change, such as perforation, from the time of admission. Based on the clinical course and examination results, postoperative ACPO was considered the fundamental cause of fulminant obstructive colitis leading to sepsis.

**Conclusions:**

ACPO following minimally invasive surgery is exceedingly rare. However, it is important to consider this disease as one of the differential diagnoses to avoid missing the chance for advanced therapy.

## Background

Acute colonic pseudo-obstruction (ACPO), also known as Ogilvie’s syndrome, is a rare condition observed in patients with some underlying diseases [1]. Although ACPO is observed in some patients who undergo invasive surgical procedures, few reports of ACPO development after minimally invasive laparoscopic surgery exist [2, 3]. We present the first report (to the best of our knowledge) of a case of ACPO with rapid septic progression after laparoscopic inguinal hernia repair.

## Case presentation

A 78-year-old man with a diagnosis of right inguinal hernia classified as II-3 according to the Japan Hernia Society classification underwent transabdominal preperitoneal hernia repair (TAPP) at our hospital. He had a history of chronic subdural hematoma evacuation at age 77 years, appendectomy during childhood, and hypertension. He had been continuously using telmisartan for hypertension; he used no other medications. During the preoperative evaluation, although premature ventricular contraction was observed on the electrical cardiogram, no evidence of asynergy or low function of the left ventricle was detected on the echocardiogram. The pulmonary function test results were within normal limits; therefore, we assessed that his cardiopulmonary function was maintained. Surgery was performed under general anesthesia using propofol, sevoflurane, rocuronium bromide, remifentanyl, fentanyl, and sugammadex sodium. We also used cefazolin sodium hydrate as a prophylactic antibiotic and acetaminophen for postoperative pain. His postoperative progress was good except for moderate subcutaneous emphysema. He was discharged on the first postoperative day. However, he returned to the emergency department with difficulty defecating and abdominal distension on the third postoperative day.

**Fig. 1:**
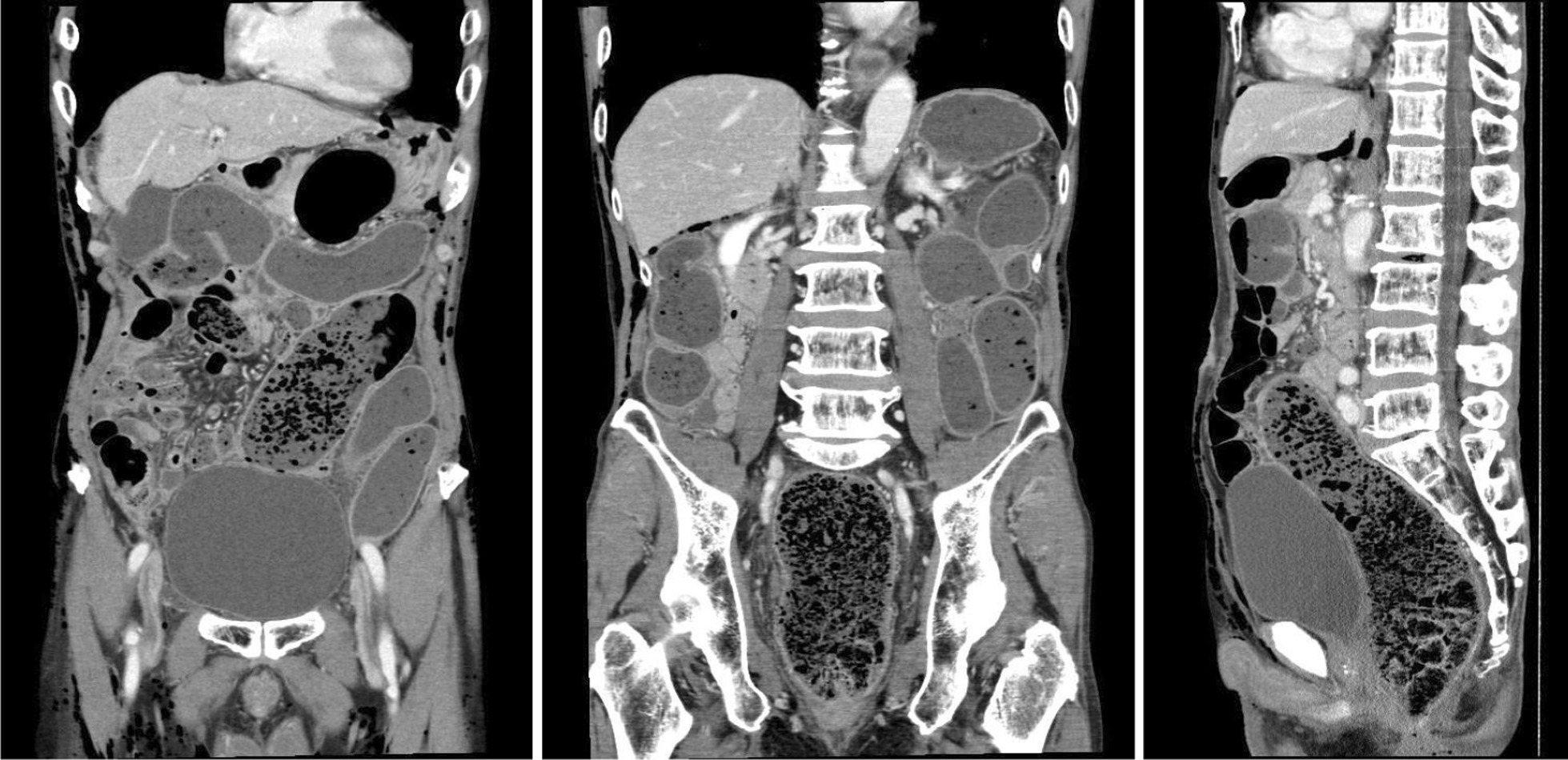
Enhanced computed tomography (CT) on admission revealed significant enlargement from the cecum to the rectum. However, small intestinal dilatation, mechanical obstruction, ischemia, and perforation were not observed

**Fig. 2:**
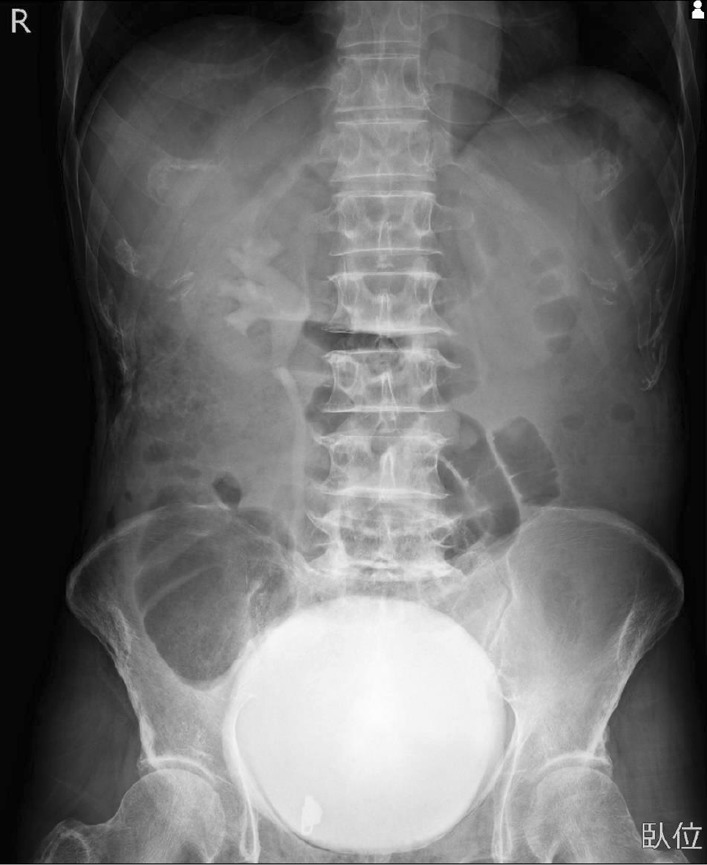
X-ray examination results 20 h after admission revealed residual radiopaque dye used for computed tomography (CT) in the dilated bladder

The patient presented with marked distension and mild tenderness of the entire abdomen without peritoneal signs; no tumor was palpable during rectal examination. His vital signs were as follows: temperature, 36.0 °C; heart rate, 80 beats per minute; and blood pressure, 160/93 mmHg. Blood test results showed a mildly elevated total white blood cell count (8600 cells/μL; neutrophils, 94.5%) and slightly increased level of C-reactive protein (0.90 mg/dL). Liver injury, renal dysfunction, and electrolyte imbalance were not detected. Enhanced computed tomography (CT) revealed significant enlargement from the cecum to the rectum (Fig. 1). The mesenteric arteries and veins were patent. The enlarged colon and rectum were well-enhanced with radiopaque dye and considered non-ischemic. There was no evidence of small intestinal dilatation, mechanical obstruction, or bowel perforation. Postoperative constipation was the first diagnosis and he was admitted for hospitalization. He received some conservative management as follows. We performed digital disimpaction, glycerin enema for colorectal decompression, and intravenous administration of panthenol to promote bowel movement; however, his nausea and vomiting did not improve. Further, we inserted a nasogastric tube for intestinal decompression and performed an X-ray examination to evaluate the tube location and bowel dilatation. Residual radiopaque dye of the CT remained in the significantly dilated bladder 20 h after admission (Fig. 2). He had also experienced dysuria after the previous discharge. We inserted a urethral catheter based on the belief that dysuria and constipation affected each other. He gradually started to release small volumes of stool, and these treatments seemed relatively successful.

**Fig. 3:**
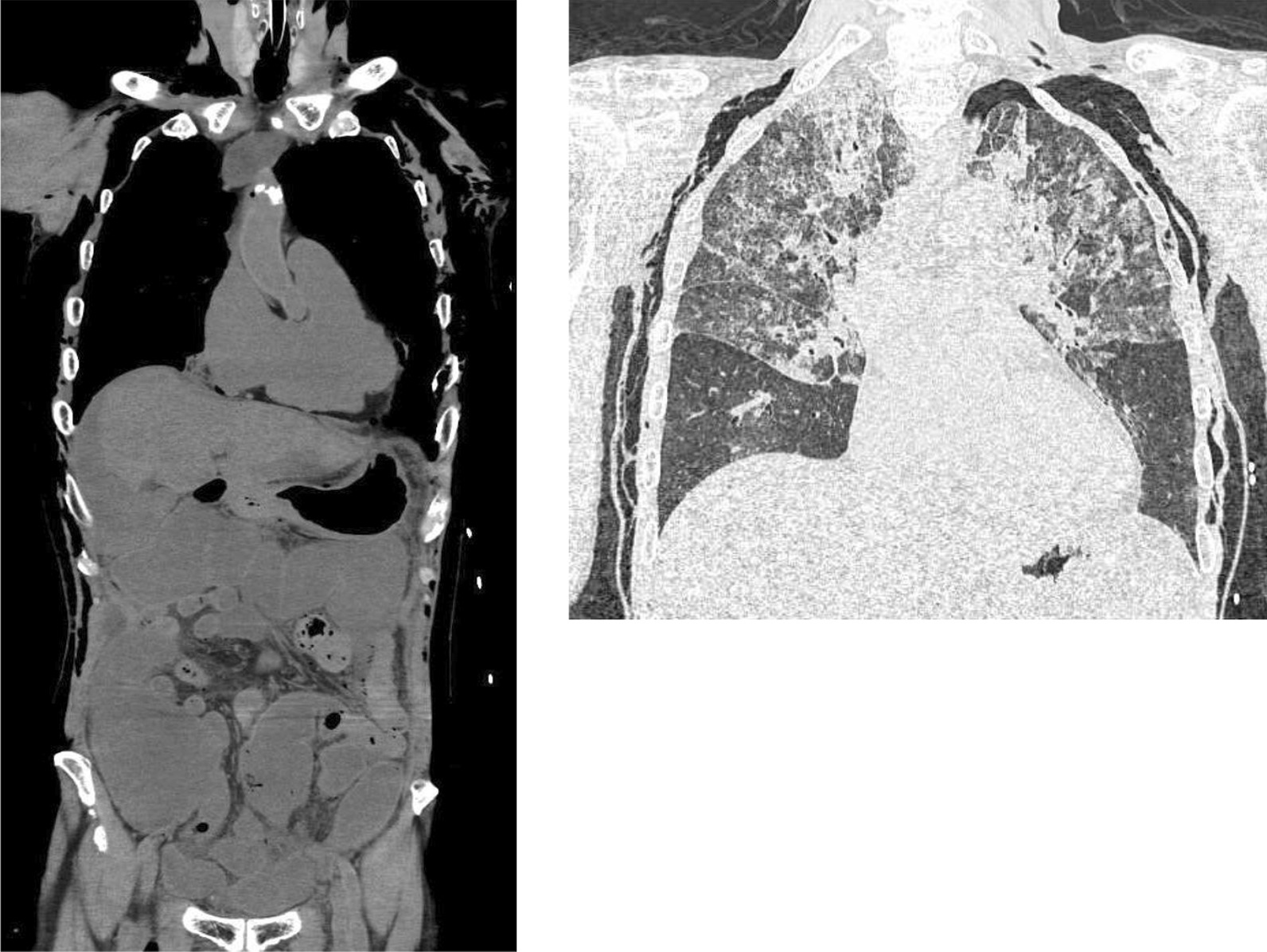
Computed tomography (CT) imaging during autopsy did not show significant changes, such as bowel perforation in the abdomen, from admission. Mild cardiomegaly, intratracheal fluid, and upper and dorsal infiltration of the lungs were observed

He later exhibited nocturnal delirium and suddenly experienced cardiopulmonary arrest 30 h after admission; he could not be resuscitated. At the time of tracheal intubation, there was no vomitus causing asphyxiation; however, there was massive foamy sputum intratracheally. Laboratory test results showed a decreased total white blood cell count (4400 cells/μL; neutrophils, 62.2%) and significantly increased level of C-reactive protein (19.27 mg/dL). The serum blood urea nitrogen (58.9 mg/dL) and creatinine (2.48 mg/dL) were also increased, indicating acute renal dysfunction. His serum troponin level was negative but D-dimer (10.9 μg/mL) was increased. CT imaging during autopsy (Fig. 3) did not show any significant change, such as bowel perforation in abdomen, from the time of admission. Cardiomegaly was not significant; however, a large quantity of intratracheal fluid and upper and dorsal infiltration of the lungs were detected. Based on the clinical course and these examination results, apart from postoperative constipation, ACPO was considered the fundamental disease and developed fulminant obstructive colitis leading to critical sepsis rapidly.

## Discussion and conclusion

ACPO, considered synonymous with Ogilvie’s syndrome, is a rare clinical state characterized by acute colonic dilatation in the absence of any mechanical obstruction [4]. This peculiar condition is primarily observed in hospitalized patients with severe illnesses such as cardiac or neurologic events, trauma, and infection, and in some who have recently undergone surgery [1]. Common symptoms are acute massive abdominal distention and pain; however, nausea, vomiting, and constipation are not consistently present [5]. Ironically, the signs of systemic toxicity do not appear until catastrophic complications have occurred. If left untreated, progressive dilation of the colon can result in colonic mural ischemia or perforation in up to 15% of cases, and the mortality rate is estimated to be 40% [1].

It is believed that functional disturbance of colonic motility leads to the development of ACPO, but the pathophysiology is not entirely clear. The most common theory used to explain this condition is an autonomic imbalance between the sympathetic and parasympathetic innervation of the colon, resulting in a relative excess of sympathetic over parasympathetic tone and leading to dilation of the colon [1]. For patients with severe underlying conditions or those who have undergone recent surgery, the sympathetic reaction might be relatively increased. Numerous medications such as anticholinergics, opiates, calcium channel blockers, and psychotropic drugs have also been associated with ACPO [1]. Patients with basic chronic illnesses might be using multiple medications that affect bowel movement; therefore, they are likely to develop ACPO. Our patient did not have a history of constipation, mental illness, or the use of any medications that could influence bowel movement during the preoperative period; therefore, his background likely did not cause ACPO. Remifentanil and fentanyl are the only perioperative medications that could be considered potential causes of ACPO; however, their effects would have already worn off at the time of re-admission. Although intraoperative injuries of the intestine or autonomic nerves during surgery were not identified retrospectively, some perioperative complications must be excluded. According to a review of 15,176 TAPP cases, the incidence rate of postoperative bowel obstruction was reported to be 0.06% [6]. There are also reports of postoperative ileus after TAPP caused by tacks [7], barbed suture devices [8], mesh erosion, and adhesions [9]. However, these complicated cases usually do not exhibit dilatation of the large intestine. Our patient also developed dilatation of bladder; therefore, it seemed that a wide range of organ hypomyotonia had occurred in the pelvis. The functions of the pelvic organs are innervated by the autonomic plexus, including the superior hypogastric plexus comprising sympathetic nerves, the inferior hypogastric plexus comprising mixed sympathetic and parasympathetic nerves, and the pelvic splanchnic nerves as parasympathetic nerves [10]. These nerves or plexus are outside the reach of operative procedures during TAPP and rarely could be injured directly. It was concluded that operative invasion itself caused the autonomic imbalance leading to colic and cystic paresis. More than approximately 20% of ACPO cases are associated with postoperative conditions such as coronary bypass, organ transplantation, cesarean delivery, and major orthopedic and supine surgery [1, 5, 11], although there are few reports of ACPO associated with laparoscopic [2, 3] and robotic-assisted [12] surgery. TAPP is considered a minimally invasive procedure; however, the incidence of this rare disease might not depend on the degree of surgical invasiveness.

Usually, the majority of patients diagnosed with ACPO are successfully managed with conservative measures such as nil per os, a nasogastric tube, erythromycin, and metoclopramide; emergent colonoscopy and surgery are rarely required [13]. Recently, the effectiveness of neostigmine as an acetylcholinesterase inhibitor to promote bowel movement has been reported [14]. If obstructive colitis is suspected as a complication, then appropriate antibiotics are also needed. These conservative measures are usually performed for several days, and more invasive procedures should be considered if the conditions worsen [5, 11].

In our patient, it was hard to determine the exact cause of sudden death. Pulmonary embolism could be raised as one of the diagnosis to be differentiated because of the increased level of D-dimer, but equivocal. Deep venous thrombosis was neither be detected in enhanced CT on admission, nor attenuation at the pulmonary trunk or main pulmonary artery in autopsy CT. A large quantity of intratracheal fluid and wide range of infiltration in lungs suggested congestion or acute pulmonary edema, however, the disease progression was too rapid to consider usual pneumonia, acute cardiac failure or even acute respiratory distress syndrome. We also assessed that his cardiopulmonary function was well-maintained according to the preoperative exams, as occurrence of a cardiovascular event was not suspicious. Based on his clinical course and all examination results, fulminant obstructive colitis and critical septic state seemed adequate for the reason of fatal cardiopulmonary injury.

Our case presented an exceptionally rapid clinical course; therefore, we missed the chance to consider surgical procedures. We could not immediately suspect ACPO because paralytic ileus seemed to be a more obvious diagnosis after surgery. However, the limited involvement of the small intestine and massive colonic dilatation, which are not typical in paralytic bowel obstruction, suggested the correct diagnosis. The interventions we performed, which comprised nasogastric tube use, digital disimpaction, and administration of a glycerin enema, were not enough to improve his condition given the distribution and pathogenesis of the disease. Monitoring his condition more carefully and using more aggressive therapy earlier, such as neostigmine or prophylactic antibiotics from the time of admission, subsequent colonoscopy for decompression, or even surgery, might have resulted in a better outcome. Furthermore, in hindsight, it could have been beneficial if we had confirmed his bacteremia by performing a blood culture test; however, his nocturnal delirium indicated critical sepsis caused by obstructive colitis.

In conclusion, our patient was a rare but highly suggestive case of ACPO after TAPP. In certain patients, such as the elderly, sepsis caused by ischemic colitis can progress rapidly. Careful observation of clinical features is indispensable to avoid a poor prognosis.

## Data Availability

Data sharing is not applicable to this article, as no datasets were generated or analyzed during the current study.

## Abbreviations

ACPO: Acute colonic pseudo-obstruction
CT: Computed tomography
TAPP: Transabdominal preperitoneal hernia repair
